# Cloning, Sequencing, and *In Silico* Analysis of **β**-Propeller Phytase *Bacillus licheniformis* Strain PB-13

**DOI:** 10.1155/2014/841353

**Published:** 2014-04-24

**Authors:** Vinod Kumar, Gopal Singh, Punesh Sangwan, A. K. Verma, Sanjeev Agrawal

**Affiliations:** ^1^Department of Biochemistry, College of Basic Sciences and Humanities, G. B. Pant University of Agriculture and Technology, Pantnagar 263145, India; ^2^Akal School of Biotechnology, Eternal University, Baru Sahib, Sirmour 173101, India; ^3^Department of Biochemistry, C. C. S. Haryana Agricultural University, Hisar 125001, India

## Abstract

**β**-Propeller phytases (BPPhy) are widely distributed in nature and play a major role in phytate-phosphorus cycling. In the present study, a BPPhy gene from *Bacillus licheniformis* strain was expressed in *E. coli* with a phytase activity of 1.15 U/mL and specific activity of 0.92 U/mg proteins. The expressed enzyme represented a full length ORF “PhyPB13” of 381 amino acid residues and differs by 3 residues from the closest similar existing BPPhy sequences. The PhyPB13 sequence was characterized *in silico* using various bioinformatic tools to better understand structural, functional, and evolutionary aspects of BPPhy class by multiple sequence alignment and homology search, phylogenetic tree construction, variation in biochemical features, and distribution of motifs and superfamilies. In all sequences, conserved sites were observed toward their N-terminus and C-terminus. Cysteine was not present in the sequence. Overall, three major clusters were observed in phylogenetic tree with variation in biophysical characteristics. A total of 10 motifs were reported with motif “1” observed in all 44 protein sequences and might be used for diversity and expression analysis of BPPhy enzymes. This study revealed important sequence features of BPPhy and pave a way for determining catalytic mechanism and selection of phytase with desirable characteristics.

## 1. Introduction

Phytases (*myo*-inositol 1,2,3,4,5,6-hexa*kis*phosphate phosphohydrolase) belongs to a special group of phosphatases which can hydrolyse phytate (*myo*-inositol 1,2,3,4,5,6-hexa*kis*phosphate, IP_6_) to inositol phosphates, inorganic phosphorus, and* myo*-inositol [1]. Phytate is synthesized by plants and represents a very significant amount of organic phosphorus (60–80%) in soil [2]. Phytase lowers down affinity of phytate to associate minerals and proteins [3] and its additions to animal feed liberate the inorganic phosphorus from bound phytate-phosphorus and make it available to the monogastric animals [4, 5].

Phytases are widely distributed among plants and microbial cells [6, 7]. Based on the specific consensus sequence, catalytic mechanism, and three dimensional structures, four classes of phytases, which have been characterized so far, are histidine acid phosphatase (HAPhy), cysteine phytase (CPhy), purple acid phosphatase (PAPhy), and beta-propeller phytase (BPPhy) [8, 9]. Alternatively, according to the initiation site of dephosphorylation of the phytate, the ENZYME database (available through the ExPASy Proteomics Server: http://enzyme.expasy.org/) classifies phytases into three groups: 3-phytase (alternative name, 1-phytase; EC 3.1.3.8), 4-phytase (alternative name, 6-phytase; EC 3.1.3.26), and 5-phytase (EC 3.1.3.72). Among them, BPPhy is widely distributed in nature and plays a major role in phytate-phosphorus cycling in both soil and aquatic microbial communities [8]. BPPhy has a six-bladed beta-propeller folding architecture [10] and dephosphorylate phytate in a stereospecific way by sequential removal of every second phosphate group. These exhibit both unique Ca^2+^-dependent catalytic property and highly strict substrate specificity for the calcium-phytate complex [11].

Bioinformatics analysis of genes and genomes from different species makes possible the identification of new genes including orthologs or paralogs [12] and also facilitates the establishment of phylogenetic relationships between genes and evolutionary molecular mechanisms [13]. Large numbers of phytase gene sequences are available in various databases providing further opportunity to study detailed mechanistic and sequential diversity of this class of enzymes. It has been utilized for formation of consensus phytase sequence [14],* in silico *analysis of HAP sequences [15], and motif analysis of different phytases [16]. However, no such study has been conducted to assess sequence diversity of BPPhy. The sequence information and further analysis of superfamily will help in understanding the underlying mechanisms and also helps to develop and/or implement a range of alternate effectors for enzyme activity. The* in silico* characterization of protein sequences of other industrially important enzymes has also been reported recently [17–19].

In the present study, a phytase producing* Bacillus licheniformis* strain was used for the isolation, cloning, and sequencing of BPPhy gene in pET32a vector and expression in* E. coli *BL21. The phytase sequence was characterized* in silico*. Simultaneously, in order to better understand the structural, functional, and evolutionary aspects of BPPhy, we exploited the reference protein sequences of BPPhy in NCBI and ExPASy databases for* in silico *study of their biochemical features, multiple sequence alignment and identity search, phylogenetic tree construction, and distribution of motifs and superfamilies using various bioinformatics tools. We provide here information regarding conserved and variable amino acids and protein motifs that might have an impact on function. In addition, we analyzed other structural aspects including the position of conserved residues and the cleavage site of the zymogen and presented a preliminary phylogenetic analysis of selected members of this subfamily.

## 2. Material and Methods

### 2.1. Chemicals and Bacterial Strains

All the chemicals, solvents, and antibiotics used in this study were of molecular biology and analytical grade and procured from standard manufacturers as GeNei, Sigma, Merck, and HiMedia Pvt. Ltd. Phytase producing* Bacillus licheniformis *strain PB-13 (identified using 16S rRNA gene sequencing, GenBank Accession number JX406744.1) isolated in our laboratory was used for isolation of phytase gene [20].* E. coli* DH5*α* and* E. coli *BL21 (DE3) (Novagen) were used as cloning host and expression host, respectively. Plasmid vector pET32a(+) (Novagen) was used for cloning and expression studies.* E. coli* strains and plasmid were kindly provided by Dr. S. P. Singh, Department of Veterinary Public Health, College of Veterinary and Animal Sciences, G. B. Pant University of Agriculture and Technology, Pantnagar.

### 2.2. PCR Cloning and Expression of the Phytase Gene

Phytase gene sequence (GenBank accession number BL018) was retrieved from complete genome sequence of* Bacillus licheniformis* ATCC 14580 from KEGG genome database (http://www.genome.jp/kegg-bin/show_organism?org=bli). Primers were designed from end regions of complete ORF. For the directional cloning, restriction sites for* Hind*III and* Xho*I were introduced at 5′ ends of forward primer, PhyL F11 “CG*AAGCTT*ATCATATGAACTTTTACAAAACG,” and reverse primer, PhyL R “GTG*CTCGAG*CCTTATTTGGCTCGTTTTTTCA,” respectively. The primers were custom-synthesized by SBS Gentech Co. Ltd. The PCR amplification was carried out using* Pfu* polymerase (Fermentas) for 30 cycles at 94°C for 45 sec, 50°C for 45 sec, and 72°C for 1 min with genomic DNA of* Bacillus licheniformis *strain PB-13 as template. For directional cloning of PCR product into pET32a(+), the amplified PCR fragment was restriction-digested with* Hind*III/*Xho*I and separated on agarose gel. The separated fragment was cut from the gel and purified with the QIAquick DNA purification kit (Qiagen). Purified* Hind*III/*Xho*I fragment was cloned into an* Hind*III/*Xho*I-cut pET32a(+)* E. coli *expression vector harbouring C-terminal His6 tag. The* E. coli *DH5*α* cells were transformed with recombinant plasmid. Recombinant plasmid from positive clone for phytase gene was isolated and transformed into expression host* E. coli* BL21- (DE3-) pLysS as per standardized protocol [21]. A colony was randomly picked from among the colonies observed on ampicillin selection plate. This was tested for presence of recombinant plasmid containing phytase gene using gene specific primers (PhyL F11 and PhyL R). The transformants were grown in LB broth containing ampicillin (100 *μ*g/mL), induced with the different amount of IPTG to optimize expression. For production analysis, samples were withdrawn at various times after induction and cells were pelleted, resuspended into 50 mM Tris-HCl buffer (pH 7.0, containing 1 mM CaCl_2_) were sonicated on ice for 5 min with a pulse rate of 30 sec and a gap of 10 sec. Cell debris were removed by centrifugation at 10000 rpm for 30 min, 4°C. The recombinant protein from supernatant was assayed for crude phytase activity.

### 2.3. Phytase Assay

Crude phytase activity was determined using 5 mM sodium phytate as substrate in 0.1 M sodium acetate buffer, with pH 5.5 following the method of Engelen et al. [22]. One unit was defined as the amount of enzyme that released 1 *μ*M of inorganic phosphate in 1 min. The amount of phosphate released was calculated based on standard curve of KH_2_PO_4_.

### 2.4. *In Silico* Analysis of B. licheniformis PB-13 Phytase Sequence

Amplified PCR products were sequenced by automated DNA sequencer at DNA Sequencing Facility, University of Delhi (South Campus), New Delhi, India. The sequence analysis was done using MEGA5 (http://www.megasoftware.net/) and NCBI database by employing BLASTN algorithm (http://blast.ncbi.nlm.nih.gov/Blast.cgi). The sequences obtained were deposited in NCBI GenBank (http://www.ncbi.nlm.nih.gov/genbank/submit/). ORF Finder (http://www.ncbi.nlm.nih.gov/projects/gorf/) was used for identifying open reading frame into gene sequence. Nucleotide sequence represented complete true ORF was translated into protein sequence using ExPASy translate tool (http://web.expasy.org/translate/) and used for* in silico* characterization. The signal peptide was predicted using SignalP (http://www.cbs.dtu.dk/services/SignalP/). The tertiary structure of rPhyPB13 was predicted using the homology modeling approach at SwissModel Workspace (http://swissmodel.expasy.org/) with the *β*-propeller phytase TS-Phy from* Bacillus amyloliquefaciens* (PDB code 1H6L) as the template [23, 24]. The evolutionary history was inferred using the neigbour-joining method [25]. The evolutionary distances were computed using the maximum composite likelihood method and are in the units of the number of base substitutions per site [26]. Evolutionary analyses were conducted in MEGA5.

### 2.5. *In Silico* Characterization of *β*-Propeller Phytase Sequence

PhyPB13 *β*-propeller phytases sequence was used as probe NCBI protein database (http://www.ncbi.nlm.nih.gov/; accessed in June, 2012) to retrieve the 44 reference protein sequences of BPPhy used in this study (Table 1). The protein sequences in FASTA format from RefSeq entries, which were shown to exhibit phytase activities, were selected for further* in silico* study. The sequences were characterized for homology, phylogenetic relationship, functional domain, and biophysical characteristics using available bioinformatic tools following methodology as adapted by Kumar et al. [15].

## 3. Result and Discussion

### 3.1. Cloning and Expression of Phytase

*E. coli* expression system is one of the simplest, cost-effective, and suitable systems for large scale production of recombinant proteins [27]. In the present study, we have used a soluble recombinant proteins expression system to express phytase from* B. licheniformis* PB-13. PCR amplification for the isolation of phytase gene resulted into an amplified PCR product of ~1,150 bp as observed after electrophoresis on 1% agarose gel. Appearance of single band on gel revealed specific amplification of phytase gene using end-specific primers. This good quality PCR product was taken for restriction digestion using* Hind*III and* Xho*I restriction enzymes.* E. coli* DH5*α* was transformed with recombinant vector (pET32a + PhyPB13 phytase gene).* E. coli* BL21 (DE3) was used as an expression host, as it encodes the T7 RNA polymerase under the control of lacUV5 promoter [28]. Transformation of plasmid from positive clone to* E. coli* BL21 competent cells followed by induction with IPTG for 4 h resulted in expression of recombinant phytase by SDS-PAGE as an intense band of ~66 kDa while no such band was observed in uninduced culture. The size of induced protein was consistent with the calculated value for the fusion protein (~63 kDa), which includes an additional peptide sequence of about 20 kDa (175 amino acids) along with encoded phytase sequence of 381 amino acids (theoretical molecular weight ~42 kDa). The additional sequence includes Trx-tag (109 amino acids; which increases solubility of expressed protein), S-tag (used in purification of recombinant proteins), His_6_-tag (role in purification), and linker sequence [28]. Despite the presence of this additional amino acid stretch, the recombinant phytase was found to be catalytically active. The recombinant phytase was designated as “rPhyPB13.” Transformed* E. coli* BL21 cells produced rPhyPB13 with an enzyme activity of 1.15 U/mL and specific activity of 0.92 U/mg proteins. It was comparable to wild type* B. licheniformis* PB-13 phytase in production media.* B. licheniformis* PB-13 produced 0.99 U/mL phytase in PSMWB media (phytase screening media supplemented with 10% wheat bran) with a specific activity of 0.70 U/mg proteins [20].

### 3.2. Sequencing and Characterization* B. licheniformis* PB-13 Phytase Gene Sequence

Sequencing of target insert from positive clone by automated DNA sequencer at Department of Biochemistry, University of Delhi (South Campus), New Delhi, resulted in a nucleotide sequence of 1,149 bp (GenBank accession number JX187608.1). Analysis of sequence using BlastN resulted into 99% identity of sequence with* B. licheniformis* phytase L precursor gene (GenBank accession number AF469936.1). The phylogenetic tree constructed using neighbor-joining method also showed similar classification.

**Figure 1:**
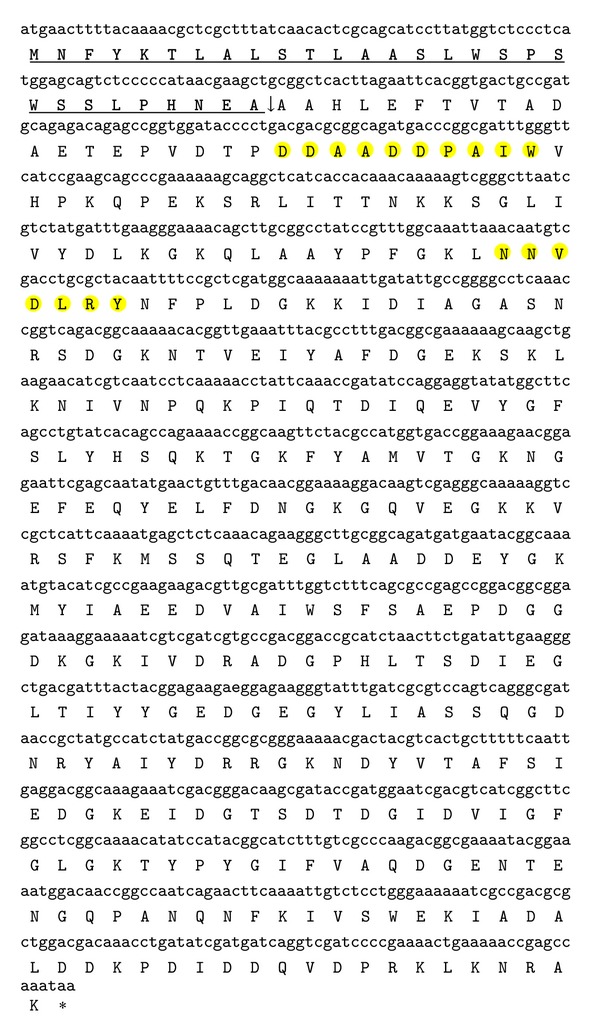
Translated protein sequence from PhyPB13 nucleotide sequence (1146 bp). Signal peptide sequence is present from amino acid residues 1–29 (sequence underlined); ↓ indicates cleavage site of signal peptide;  *asterisk indicates stop codon; conserved sequences are highlighted.

The nucleotide sequence was searched for open reading frame (ORF) using ORF Finder. Ten (10) ORFs of varying length starting from different frames were obtained. The largest sequence was present in frame +1 which corresponded to the true ORF for phytase gene as it was, which started from first nucleotide and ended with a stop codon. Also, it showed 99% similarity to phytase sequences present in GenBank database. This full length ORF designated as “PhyPB13” encoded a protein of 381 amino acid residues with a calculated molecular mass of 42.1 kDa. The nucleotide sequence along with translated protein sequence (GenBank accession number AFQ59979.1) using ExPASy translation tool contained a putative signal peptide of 29 amino acid residues starting from amino acid residue 1 to 29. A cleavage site was present between residues 29 and 30 (Figure 1). Wang et al. [29] isolated a* phyC* gene of 1,146 bp from* B. licheniformis *encoding a peptide of 381 amino acids. The length of signal peptide in* phyC* was 31 amino acids. A BPPhy gene with an ORF of 1,074 bp (357 amino acid residues) and a signal peptide of 27 amino acid residues was isolated from* P. nyakensis* [30]. The amino acid composition of PhyPB13 protein sequence determined using ProtParam server revealed that Asp, Gly, Lys, and Ala were major amino acids constituting about 36% of PhyPB13. Cysteine was not observed in the sequence indicating that PhyPB13 did not bear disulfide bonds, which were believed to be essential for conformational stability and catalytic activity in several fungal phytases [29, 31, 32]. It was consistent with absence of cysteine in phytase from* B. licheniformis* [29].

Alignment of homologous sequences with Mega5 revealed presence of two conserved motifs, namely, “D-A-[A/T/E]-D-D-P-A-[I/L/V]-W” (amino acids 51–59) and “N-N-[V/I]-D-[I/L/V]-R-[Y/D/Q]” (amino acids 98–104), in PhyPB13 and other homologous sequences (Figure 1). Similar motifs were reported in multiple sequence alignments of 66 BPPhy sequences by Huang et al. [30]. Like other* Bacillus *phytases, PhyPB13 did not show sequence homology with HAPhys. The conserved regions “RHGXRXP” and “HD” of HAPhys [33] were absent in PhyPB13. Functional domain analysis using pfam (http://www.sanger.ac.uk/resources/software/) showed that the complete sequence (residues 1–381) was encoding a phytase enzyme. The sequence (residues 34–378) belongs to a thermostable phytase (3-phytase) superfamily (ID 50956) as indicated by Superfam (http://supfam.cs.bris.ac.uk/SUPERFAMILY/hmm.html) analysis. This superfamily includes thermostable phytases such as phytase from* B. amyloliquefaciens* and the other* Bacillus* sp. with 6-bladed beta-propeller fold structure. A putative conserved domain of phytase superfamily has been detected while performing a BlastP (http://blast.ncbi.nlm.nih.gov/Blast.cgi?PAGE=Proteins) similarity search analysis of PhyPB13 protein sequence. Further, the sequence appeared to be 99% identical to* phyL* precursor from* B. licheniformis* (GenBank accession number AAM74021.1). Alignment of PhyPB13 with* phyL* precursor sequence revealed that the sequences were different at three positions (PhyPB13 contains Leu, Lys, and Asn in place of Lys, Asp, and Asp at 33rd, 67th, and 281st positions, resp.).

### 3.3. Prediction of Three-Dimensional Structure of PhyPB13

Analysis of suitable template for 3D structure model of PhyPB13 using Phyre2 server (http://www.sbg.bio.ic.ac.uk/phyre2/html/page.cgi?id=index) revealed* B. amyloliquefaciens* phytase (TS-Phy, PDB ID—1H6L) as the best template for 3D modeling based on number of aligned residues and quality of alignment, with a “confidence” score of 100% which indicated the probability that a match between PhyPB13 and TS-Phy was based on homology. A match with “confidence >90%” represents similar fold and high accuracy in the modeling of core protein. The identity between target sequence and template was ~68%, which revealed accuracy of model; as for extremely high accuracy models this number should be above 30–40% (http://www.sbg.bio.ic.ac.uk/phyre2/html/help.cgi?id=help). Tridimensional structure of TS-Phy was downloaded from PDB (PDB ID 1H6L) and its PDB ID was provided as template for 3D structure prediction of PhyPB13 protein sequence using SWISS-Model server. It features automated modeling of homooligomeric assemblies and modeling of essential metal ions and cofactors in protein structures [23, 24]. Small E-value in sequence identity indicates that the TS-Phy and rPhyPB13 have a very similar sequence and good reliability of the alignment. The model has a six-bladed-propeller folding architecture [10] and 7 calcium binding sites in protein sequence predicted by 3DLigandSite [34]. Oh et al. [35] reported that an electronegative central channel accessible to solvent binds seven Ca^2+^ ions and these Ca^2+^ ions have been found important in catalytic activity and substrate binding of BPPhy. Valine at 100th position was found to be a putative ligand binding site with 4 contacts as predicted by 3DLigandSite [34]. It is present inside of the conserved region of BPPhys (residues 98–104) and might play an important role in the binding of substrate for enzyme catalysis.

### 3.4. *In Silico* Analysis and Characterization of BPPhy

The accession numbers along with source organisms of 44 reference protein sequences of BPPhy are given in Table 1. The majority of the sequences were reported to be from bacterial species dominated by* Bacillus* and* Paenibacillus* species (17 sequences). Analysis of multiple sequence alignment revealed the presence of conserved regions throughout the sequences. In all sequences, a conserved site “[D/A][STA]DDPA[I/V]W[I/V/L]T[N/D/L]K” was observed toward their N-terminus, followed by one more sequence “NN[F/V]D[I/V/L].” Huang et al. [30] reported the presence of similar sequences “DA[A/T/E]DDPA[I/L/V]W” and “NN[V/I] D[I/L/V]R[Y/D/Q]” with minor differences (sequence information was not given) during analysis of several BPPhy sequences. In the present study, we have also observed the presence of highly conserved sequence “DG” towards its C-terminus. Aspartic acid at conserved C-terminal “DG” sequence in these BPPhy sequences might act as a proton donor to the oxygen atom of the scissile phosphomonoester bond and may play a role in catalytic mechanism of these enzymes. Similar role has been suggested for aspartic acid in conserved “HD” residues towards C-terminal in HAPhy sequences [36, 37].

**Figure 2:**
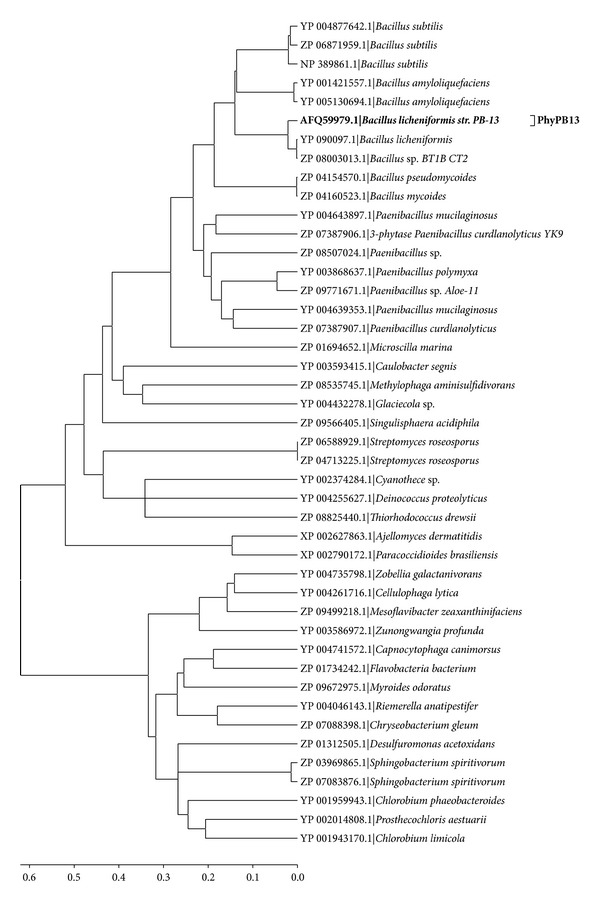
Phylogenetic tree of PhyPB13 with BPPhy protein sequences constructed by Neighbor-Joining method.

Evolutionary relationship among different sequences was studied using phylogenetic tree constructed by neighbor-joining method (Figure 2). Overall, three major clusters were observed in phylogenetic tree. Cluster “1” represented sequences of* Bacillus* with* Paenibacillus* species. The amino acid residues in sequences of this cluster were 380 ± 10 except for three sequences from* Paenibacillus* sp., that is, Y_003868637.1, YP_004639353.1, and ZP_07387907.1 which have length of 465, 461, and 469 amino acid residues, respectively. Cluster “2” represents BPPhy with the largest protein sequence in the range of 436–769, while cluster “3” had the smallest sequence with 331 to 364 amino acid residues (Table 2).

Other biophysical features of all protein sequences are also given in Table 2. Molecular weight of sequences varied according to length of protein sequences in the range of 37–82 kDa. Isoelectric point (pI) was found between 4.1 and 6.4 with the majority of sequences having a pI value above 5. The pI values for the sequences were highest in cluster 2, followed by cluster 1 and 3, respectively. The instability index was used to measure* in vivo* half-life of a protein [38]. Analysis of instability index indicated uniformity among all sequences of BPPhy and was predicted to be below 40 for all sequences except phytase from* C. phaeobacteroides* (YP_001959943.1). Further, a majority of sequences have instability index less than 30, suggesting that these proteins exhibited good* in vivo* stability [38]. Aliphatic index of reported protein sequences ranged from 69 to 90, indicating the high thermostability of BPPhy enzymes. Aliphatic index of protein measures the relative volume occupied by aliphatic side chains of the amino acids: alanine, valine, leucine, and isoleucine. Globular proteins with high aliphatic index have high thermostability and an increase in aliphatic index suggests an increase in protein thermostability [39]. Superfam server based analysis of protein sequences revealed their similarity to thermostable phytase (3-phytase) superfamily (Table 3). This family represents phytases which are thermostable at high temperatures and have a distinct catalytic mechanism with removal of initial phosphorus from 3rd carbon of phytate ring. A total of 10 motifs with given parameters were reported by MEME analysis. The 29 amino acid residues long motif “1” “DDPAIWVHPHDPEKSRIIGTNKKSGLAVY” was observed in all 44 protein sequences, with a conserved sequence “DDPAIW[VI][HN]PK[DN]P[ESA]KS.” This sequence might be used for diversity and expression analysis of BPPhy enzymes. Functional domain analysis using BlastP search for this motif revealed that the sequence belongs to phytase superfamily (Table 4).

## 4. Conclusion

In conclusion, a *β*-propeller phytase of 3-phytase family from* B. licheniformis* strain PB-13 was successfully expressed in* E. coli* BL21. Phylogenetic clustering, conserved motifs sequences, and variation among biochemical features of different BPPhy phytases in this study could be key information for screening of novel phytases and comparison with other classes of phytases, which might contribute in further classification and application of diverse BPPhys. Functional characterization of amino acid residues in conserved regions of BPPhy is required for determining their role in enzyme catalysis. Overall, this* in silico* analysis will be important for future genetic engineering of this most diverse and important class of phytase.

## Figures and Tables

**Table 1:** List of source organisms of retrieved BPPhy protein sequences (with accession number).

S. number	Source organism	Accession number	Total sequences
1	*Sphingobacterium spiritivorum *	ZP_03969865.1, ZP_07083876.1	2
2	*Desulfuromonas acetoxidans *	ZP_01312505.1	1
3	*Capnocytophaga canimorsus *	YP_004741572.1	1
4	*Chlorobium phaeobacteroides *	YP_001959943.1	1
5	*Prosthecochloris aestuarii *	YP_002014808.1	1
6	*Myroides odoratus *	ZP_09672975.1	1
7	*Riemerella anatipestifer *	YP_004046143.1	1
8	*Flavobacteria bacterium *	ZP_01734242.1	1
9	*Chlorobium limicola *	YP_001943170.1	1
10	*Zobellia galactanivorans *	YP_004735798.1	1
11	*Chryseobacterium gleum *	ZP_07088398.1	1
12	*Cellulophaga lytica *	YP_004261716.1	1
13	*Mesoflavibacter zeaxanthinifaciens *	ZP_09499218.1	1
14	*Zunongwangia profunda *	YP_003586972.1	1
15	*Cyanothece sp. *	YP_002374284.1	1
16	*Paenibacillus mucilaginosus *	YP_004643897.1, YP_004639353.1	2
17	*Paenibacillus curdlanolyticus *	ZP_07387906.1, ZP_07387907.1	2
18	*Paenibacillus polymyxa *	YP_003868637.1	1
19	*Paenibacillus sp. *	ZP_08507024.1, ZP_09771671.1	2
20	*Bacillus pseudomycoides *	ZP_04154570.1	1
21	*Bacillus mycoides *	ZP_04160523.1	1
22	*Singulisphaera acidiphila *	ZP_09566405.1	1
23	*Bacillus subtilis *	YP_004877642.1, ZP_06871959.1, NP_389861.1	3
24	*Bacillus licheniformis *	YP_090097.1, **AFQ59979.1 **	2
25	*Streptomyces roseosporus *	ZP_06588929.1, ZP_04713225.1	2
26	*Ajellomyces dermatitidis *	XP_002627863.1	1
27	*Deinococcus proteolyticus *	YP_004255627.1	1
28	*Bacillus sp. *	ZP_08003013.1	1
29	*Microscilla marina *	ZP_01694652.1	1
30	*Paracoccidioides brasiliensis *	XP_002790172.1	1
31	*Caulobacter segnis *	YP_003593415.1	1
32	*Bacillus amyloliquefaciens *	YP_001421557.1, YP_005130694.2	2
33	*Methylophaga aminisulfidivorans *	ZP_08535745.1	1
34	*Glaciecola sp. *	YP_004432278.1	1
35	*Thiorhodococcus drewsii *	ZP_08825440.1	1

**Table 2:** Biochemical characteristics of BPPhy protein sequences determined by ProtParam server.

S. number	Accession number	Source organisms	Number of amino acids	Molecular weight	Theoretical pI	Instability index	Aliphatic index
1	ZP_03969865.1	*Sphingobacterium spiritivorum *	362	40320.5	5.74	30.35	81.88
2	ZP_07083876.1	*Sphingobacterium spiritivorum *	362	40216.4	5.74	30.79	81.35
3	ZP_01312505.1	*Desulfuromonas acetoxidans *	364	39756.6	4.8	24.84	83.08
4	YP_004741572.1	*Capnocytophaga canimorsus *	343	38361.5	5.02	28.98	86.41
5	YP_001959943.1	*Chlorobium phaeobacteroides *	356	39458.3	5.34	41.08	84.1
6	YP_002014808.1	*Prosthecochloris aestuarii *	352	38123.9	5.05	27.67	85.65
7	ZP_09672975.1	*Myroides odoratus *	355	39587.8	5.04	33.9	83.58
8	YP_004046143.1	*Riemerella anatipestifer *	347	38778.8	6.34	28.8	83.4
9	ZP_01734242.1	*Flavobacteria bacterium *	355	39803.3	6.48	24.05	89.48
10	YP_001943170.1	*Chlorobium limicola *	352	38025.1	5.62	26.4	88.86
11	YP_004735798.1	*Zobellia galactanivorans *	338	37881.9	4.89	27.27	81.04
12	ZP_07088398.1	*Chryseobacterium gleum *	350	39037.2	5.46	28.92	84.03
13	YP_004261716.1	*Cellulophaga lytica *	339	37698.9	6.24	25.59	82.45
14	ZP_09499218.1	*Mesoflavibacter zeaxanthinifaciens *	337	37423.4	4.83	28.07	80.68
15	YP_003586972.1	*Zunongwangia profunda *	331	37122.4	4.6	29.72	70.06
16	YP_002374284.1	*Cyanothece *sp.* PCC 8801 *	436	46836.3	4.22	23.75	91.88
17	AFQ59979.1	*Bacillus licheniformis PB-13 *	381	42131.5	4.74	25.94	69.95
18	YP_004643897.1	*Paenibacillus mucilaginosus *	390	41788.1	4.21	22.42	86.85
19	ZP_07387906.1	*Paenibacillus curdlanolyticus *	371	40205.9	4.1	21.3	81.75
20	YP_003868637.1	*Paenibacillus polymyxa *	465	50676.9	4.93	22.81	81.83
21	YP_004639353.1	*Paenibacillus mucilaginosus *	461	49436.7	4.34	30.42	83.45
22	ZP_08507024.1	*Paenibacillus *sp.* HGF7 *	462	49590.4	4.92	17.07	82.19
23	ZP_04154570.1	*Bacillus pseudomycoides *	390	42684.7	5.34	18.5	78.26
24	ZP_04160523.1	*Bacillus mycoides *	390	42698.7	5.34	18.28	78.51
25	ZP_09566405.1	*Singulisphaera acidiphila *	366	39065.5	5.19	32.19	80.49
26	ZP_07387907.1	*Paenibacillus curdlanolyticus *	469	51012.5	5.22	22.09	88.44
27	YP_004877642.1	*Bacillus subtilis *subsp.* Spizizenii *	382	41965.4	5.19	16.27	74.55
28	YP_090097.1	*Bacillus licheniformis ATCC 14580 *	381	42040.6	4.81	26.14	70.73
29	ZP_06871959.1	*Bacillus subtilis *	382	41896.4	5.2	15.89	83.72
30	ZP_09771671.1	*Paenibacillus *sp.* Aloe-11 *	465	50835.1	5.13	21.29	82.04
31	ZP_06588929.1	*Streptomyces roseosporus *	436	46575.8	4.24	29.17	76.31
32	XP_002627863.1	*Ajellomyces dermatitidis *	768	81904.9	4.81	36.48	80.01
33	ZP_04713225.1	*Streptomyces roseosporus *	442	47136.5	4.24	29.91	76.61
34	YP_004255627.1	*Deinococcus proteolyticus *	381	40092.9	4.61	35.16	89.82
35	NP_389861.1	*Bacillus subtilis str. 168 *	382	41946.4	5.1	20.24	74.55
36	ZP_08003013.1	*Bacillus *sp.* * *BT1B CT2 *	381	42245.8	4.81	28.46	71.23
37	ZP_01694652.1	*Microscilla marina *	392	43056.2	5.09	24.76	75.61
38	XP_002790172.1	*Paracoccidioides brasiliensis *	769	81961.4	5.64	28.65	85.18
39	YP_003593415.1	*Caulobacter segnis *	673	70502.5	5.25	26.88	91.62
40	YP_001421557.1	*Bacillus amyloliquefaciens *	383	41723.3	5.02	23.7	71.91
41	ZP_08535745.1	*Methylophaga aminisulfidivorans *	640	70716	5.06	29.58	90.81
42	YP_004432278.1	*Glaciecola *sp.	656	71676.7	4.78	33.13	96.33
43	YP_005130694.1	*Bacillus amyloliquefaciens *	383	41812.3	5.07	24.87	69.87
44	ZP_08825440.1	*Thiorhodococcus drewsii *	762	82173.3	4.22	34.55	88.82

**Table 3:** Distribution of superfamily among BPPhy determined using superfam server.

Superfamily	Family	Accession number (range of amino acids residues)
Thermostable phytase (3-phytase)	Thermostable phytase (3-phytase)	YP_004767129.1 (35–378), **AFQ59979.1 (PhyPB13)** (34–375), YP_001421557.1 (31–379), YP_005130694.1 (31–379), ZP_08003013.1 (35–378), YP_004877642.1 (34–378), NP_389861.1 (35–378), ZP_06871959.1 (34–378), ZP_04154570.1 (44–383), ZP_04160523.1 (44–383), ZP_01694652.1 (56–392), YP_003868637.1 (120–461), ZP_09771671.1 (120–461), ZP_08507024.1 (118–457), ZP_07387906.1 (28–368), YP_004639353.1 (119–445), ZP_08535745.1 (59–281), YP_003593415.1 (31–339), ZP_07387907.1 (121–465), YP_004643897.1 (40–384), YP_004432278.1 (60–281), XP_002790172.1 (402–735), YP_002374284.1 (23–402), XP_002627863.1 (402–734), YP_004741572.1 (25–342), ZP_09566405.1 (28–354), YP_001959943.1 (28–352), YP_002014808.1 (32–350), ZP_01312505.1 (44–360), ZP_03969865.1 (49–355), ZP_07083876.1 (49–355), ZP_07088398.1 (36–337), ZP_01734242.1 (31–351), YP_004046143.1 (33–337), YP_001943170.1 (30–346), ZP_08825440.1 (409–761), ZP_09499218.1 (22–331), YP_003586972.1 (11–320), ZP_06588929.1 (21–284, 312–434), ZP_04713225.1 (27–290, 318–440), YP_004735798.1 (20–332), YP_004255627.1 (40–378), YP_004261716.1 (25–333), ZP_09672975.1 (26–345)

**Table 4:** Distribution of commonly observed motifs in different BPPhy protein sequences along with their functional domains.

Motifs	Motif width	Motif present in number of sequence	Amino acid sequence	Conserved region for degenerate primers	Domain
1	29	44	DDPAIWVHPHDPEKSRIIGTNKKSGLAVY	DDPAIW[VI][HN]PK[DN]P[ESA]KS	Phytase superfamily
2	30	43	QIEGCVADDEYGYMYIAEEQHCIWKYYAEP	[AV]DDE[YL]GY[LIV]Y	Phytase superfamily
3	24	43	GYLMVSSQGNNSYAIYERQGNNRY	GY[LI][IL][AV]SSQ	Local conserved
4	30	33	IDGTSETDGIDVMGFGLGPKFPHGIFVAQD	IDG[TV]S[DE][TS]DGIDV	Local conserved
5	33	21	EVYGFCLYHSQKTGKFYAMVTGKEGEFEQYELF	EVYGFSLYHS[QL]KTGK[FY]YA[LM]V[TL]GKEGEFEQYELF	Local conserved
6	16	44	RMNNVDVRYGFPLNGK	NNVD[VLI]RY[GD]F	Local conserved
7	21	44	FDGEHFTADHEGLTIYYGPDG	GEH[LF]TAD[IV]EG[LI]	Local conserved
8	29	22	GENMDHGQKVNQNFKMVPWERIAQHFPRP	K[AV]NQNFK[IM]V	Local conserved
9	15	43	KIDIAAATNRSTNKI	K[VIT]D[IL]A[AV][AV][TS][NE]RST[NG][KT][ILV]	Local conserved
10	15	42	GQITGKLVREFKMWS	G[KQ][VI]T[GA][KT][LK]VR[EK]F[KG]	Local conserved

## References

[1] Wyss M, Brugger R, Kronenberger A (1999). Biochemical characterization of fungal phytases (*myo*-inositol hexakisphosphate phosphohydrolases): catalytic properties. *Applied and Environmental Microbiology*.

[2] Lim BL, Yeung P, Cheng C, Hill JE (2007). Distribution and diversity of phytate-mineralizing bacteria. *ISME Journal*.

[3] Mitchell DB, Vogel K, Weimann BJ, Pasamontes L, Van Loon APGM (1997). The phytase subfamily of histidine acid phosphatases: Isolation of genes for two novel phytases from the fungi *Aspergillus terreus* and *Myceliophthora thermophila*. *Microbiology*.

[4] Rutherfurd SM, Chung TK, Moughan PJ (2002). The effect of microbial phytase on ileal phosphorus and amino acid digestibility in the broiler chicken. *British Poultry Science*.

[5] Olukosi OA, Cowieson AJ, Adeola O (2007). Age-related influence of a cocktail of xylanase, amylase, and protease or phytase individually or in combination in broilers. *Poultry Science*.

[6] Hegeman CE, Grabau EA (2001). A novel phytase with sequence similarity to purple acid phosphatases is expressed in cotyledons of germinating soybean seedlings. *Plant Physiology*.

[7] Kim Y-O, Kim H-K, Bae K-S, Yu J-H, Oh T-K (1998). Purification and properties of a thermostable phytase from *Bacillus sp.* DS11. *Enzyme and Microbial Technology*.

[8] Lei XG, Porres JM, Mullaney EJ, Brinch-Pedersen H (2007). *Phytase: Source, Structure and Application*.

[9] Mullaney EJ, Ullah AHJ (2007). *Phytases: Attributes, Catalytic Mechanisms and Applications. Inositol Phosphates: Linking Agriculture and the Environment*.

[10] Ha N-C, Oh B-C, Shin S (2000). Crystal structures of a novel, thermostable phytase in partially and fully calcium-loaded states. *Nature Structural Biology*.

[11] Fu S, Sun J, Qian L, Li Z (2008). *Bacillus* phytases: present scenario and future perspectives. *Applied Biochemistry and Biotechnology*.

[12] Roelofs J, Van Haastert PJM (2001). Genes lost during evolution. *Nature*.

[13] Salzberg SL, White O, Peterson J, Eisen JA (2001). Microbial genes in the human genome: lateral transfer or gene loss?. *Science*.

[14] Lehmann M, Pasamontes L, Lassen SF, Wyss M (2000). The consensus concept for thermostability engineering of proteins. *Biochimica et Biophysica Acta, Protein Structure and Molecular Enzymology*.

[15] Kumar V, Singh G, Verma AK, Agrawal S (2012). *In Silico* characterization of histidine acid phytase sequences. *Enzyme Research*.

[16] Fan CM, Wang YH, Fu CY, Zheng YF (2013). Fingerprint motifs of phytases. *African Journal of Biotechnology*.

[17] Dubey AK, Yadav S, Kumar M, Singh VK, Sarangi BK, Yadav D (2010). *In Silico* characterization of pectate lyase protein sequences from different source organisms. *Enzyme Research*.

[18] Malviya N, Srivastava M, Diwakar SK, Mishra SK (2011). Insights to sequence information of polyphenol oxidase enzyme from different source organisms. *Applied Biochemistry and Biotechnology*.

[19] Morya VK, Yadav S, Kim E-K, Yadav D (2012). In silico characterization of alkaline proteases from different species of aspergillus. *Applied Biochemistry and Biotechnology*.

[20] Kumar P (2010). *Production and characterization of bacterial phytase and its assessment as feed additive [Ph.D. thesis]*.

[21] Sambrook J, Russell DW (2001). *Molecular Cloning: A Laboratory Manual*.

[22] Engelen AJ, van der Heeft FC, Randsdorp PH, Smit EL (1994). Simple and rapid determination of phytase activity. *Journal of AOAC International*.

[23] Arnold K, Bordoli L, Kopp J, Schwede T (2006). The SWISS-MODEL workspace: a web-based environment for protein structure homology modelling. *Bioinformatics*.

[24] Guex N, Peitsch MC (1997). SWISS-MODEL and the Swiss-PdbViewer: an environment for comparative protein modeling. *Electrophoresis*.

[25] Saitou N, Nei M (1987). The neighbor-joining method: a new method for reconstructing phylogenetic trees. *Molecular biology and evolution*.

[26] Tamura K, Nei M, Kumar S (2004). Prospects for inferring very large phylogenies by using the neighbor-joining method. *Proceedings of the National Academy of Sciences of the United States of America*.

[27] Demain AL, Vaishnav P (2009). Production of recombinant proteins by microbes and higher organisms. *Biotechnology Advances*.

[28] Zhou Q-F, Luo X-G, Ye L, Xi T (2007). High-level production of a novel antimicrobial peptide perinerin in *Escherichia coli* by fusion expression. *Current Microbiology*.

[29] Wang Q, Fu S-J, Sun J-Y, Weng X-Y (2011). Characterization of a thermostable alkaline phytase from *Bacillus licheniformis* ZJ-6 in *Pichia pastoris*. *World Journal of Microbiology and Biotechnology*.

[30] Huang H, Shao N, Wang Y (2009). A novel beta-propeller phytase from *Pedobacter nyackensis* MJ11 CGMCC 2503 with potential as an aquatic feed additive. *Applied Microbiology and Biotechnology*.

[31] Song GY, Wang XY, Wang M (2005). Influence of disulfide bonds on the conformational changes and activities of refolded phytase. *Protein and Peptide Letters*.

[32] Ullah AHJ, Mullaney EJ (1996). Disulfide bonds are necessary for structure and activity in *Aspergillus ficuum* phytase. *Biochemical and Biophysical Research Communications*.

[33] Van Etten RL, Davidson R, Stevis PE, MacArthur H, Moore DL (1991). Covalent structure, disulfide bonding, and identification of reactive surface and active site residues of human prostatic acid phosphatase. *The Journal of Biological Chemistry*.

[34] Wass MN, Kelley LA, Sternberg MJE (2010). 3-D LigandSite: predicting ligand-binding sites using similar structures. *Nucleic Acids Research*.

[35] Oh B-C, Kim MH, Yun B-S (2006). Ca^2+^-inositol phosphate chelation mediates the substrate specificity of *β*-propeller phytase. *Biochemistry*.

[36] Lindqvist Y, Schneider G, Vihko P (1994). Crystal structures of rat acid phosphatase complexed with the transition-state analogs vanadate and molybdate. Implications for the reaction mechanism. *European Journal of Biochemistry*.

[37] Porvari KS, Herrala AM, Kurkela RM (1994). Site-directed mutagenesis of prostatic acid phosphatase. Catalytically important aspartic acid 258, substrate specificity, and oligomerization. *The Journal of Biological Chemistry*.

[38] Guruprasad K, Reddy BVB, Pandit MW (1990). Correlation between stability of a protein and its dipeptide composition: a novel approach for predicting *in vivo* stability of a protein from its primary sequence. *Protein Engineering*.

[39] Ikai A (1980). Thermostability and aliphatic index of globular proteins. *Journal of Biochemistry*.

